# Facing the crowd: intruder pressure, within-group competition, and the resolution of conflicts over group-membership

**DOI:** 10.1002/ece3.533

**Published:** 2013-03-19

**Authors:** Markus Port, Rufus A Johnstone

**Affiliations:** 1 Courant Research Center Evolution of Social Behavior, Research Group Social Evolution in Primates, University of GöttingenKellnerweg 6, 37077, Göttingen, Germany; 2 Centre for Ecology and Conservation, College of Life and Environmental Sciences, University of ExeterCornwall Campus, Penryn, TR10 9E, United Kingdom; 3 Department of Zoology, University of CambridgeDowning Street, Cambridge, CB2 3EJ, United Kingdom

**Keywords:** Conflict resolution, group-living, reproductive skew, social evolution, territoriality

## Abstract

Recent theory in social evolution has been mainly concerned with competition and cooperation within social groups of animals and their impact on the stability of those groups. Much less attention has been paid to conflicts arising as a result of solitary floaters (outsiders) attempting to join groups of established residents (insiders). We model such conflicts over group-membership using a demographically explicit approach in which the rates of births and deaths in a population determine the availability of group-vacancies and the number of floaters competing over these vacancies. We find that the outcome of within-group competition, reflected in the partitioning of reproduction among group members, exerts surprisingly little influence on the resolution of insider-outsider conflict. The outcome of such conflict is also largely unaffected by differences in resource holding potential between insiders and outsiders. By contrast, whether or not groups form is mainly determined by demographic factors (variation in vital rates such as fecundity and mortality) and the resulting population dynamics. In particular, at high floater densities territory defense becomes too costly, and groups form because insiders give in to the intruder pressure imposed on them by outsiders. We emphasize the importance of insider-outsider conflicts in social evolution theory and highlight avenues for future research.

## Introduction

Living in groups is generally associated with benefits such as protection from predators, increased foraging efficiency, or communal defense of resources (Krause and Ruxton 2002). But group-living can also incur costs owing to within-group competition over resources or mates (Pulliam and Caraco 1984; Krause and Ruxton 2002). If the costs associated with an increase in group size exceed the benefits, established group-members or territory owners (insiders) may do best to repel potential entrants to the group or territory. Yet in this situation, non members (outsiders) may still benefit from joining if group-living yields higher fitness gains than remaining solitary. A conflict over group membership thus arises between insiders and outsiders, and whether or not groups form, and the size they attain, depends on the outcome of this conflict (Giraldeau and Caraco 1993; Higashi and Yamamura 1993). This type of conflict is of particular importance for the evolution of vertebrate mating systems (Emlen and Oring 1977; Clutton-Brock 1989; Kappeler and van Schaik 2002). For instance, in dunnocks *(Prunella modularis*, Davies 1992) or speckled warblers (*Chthonicola sagittata*, Gardner et al. 2003) males do best by living in monogamous or polygynous breeding-groups, but often cannot prevent floaters from settling on their territories. Likewise, in a Malagasy lemur (*Propithecus verreauxi*), despite the absence of synergistic benefits, males occasionally live in multi-male associations, presumably because outsiders manage to force themselves into established groups (Port et al. 2012).

It is perhaps surprising that, despite the empirical significance of insider-outsider conflict, only a few theoretical studies have so far examined this issue (Pulliam and Caraco 1984; Giraldeau and Caraco 1993; Higashi and Yamamura 1993). These models have acknowledged that the strength of selection on outsiders to gain group membership depends on their fitness prospects as solitary individuals. However, they have generally treated these fitness prospects as a fixed parameter, and have not focused on how the fitness of a solitary individual depends on the dynamics of the population it belongs to. For instance, it should depend on the degree of habitat saturation (Kokko and Lundberg 2001), which is affected by the behavior of the outsiders themselves.

In recent years, several demographically explicit models of social evolution have been developed that allow for such feedbacks between individual behavior and population dynamics (Pen and Weissing 2000; Kokko and Lundberg 2001; Kokko and Ekman 2002; Hamilton and Taborsky 2005; Port et al. 2011). These models focus on the process of group formation either through delayed dispersal (where ‘outsiders’ are offspring remaining on their natal patch; Pen and Weissing 2000; Kokko and Lundberg 2001; Kokko and Ekman 2002; Hamilton and Taborsky 2005) or through immigration into groups of non-relatives (Port et al. 2011). Implicitly, however, most models assume either, that groups are formed via free entry of outsiders (i.e., complete outsider control; Pen and Weissing 2000; Kokko and Ekman 2002), or that insiders can reject (or evict) outsiders at no cost (i.e., complete insider control; Kokko and Lundberg 2001; Port et al. 2011). This approach is appropriate as long as both parties benefit from group-living, but it leaves open the question of how possible conflicts over group-membership are resolved.

In the present study, we therefore synthesize both lines of research. We develop a demographically explicit model of social evolution which incorporates an explicit analysis of insider-outsider conflict over group membership. We focus on a situation in which outsiders benefit from joining a group, but in which insiders do not benefit from sharing group-membership. We ask how vital rates, such as fecundity and mortality affect the turnover rate of territories in the population. We then examine (i) how the availability of vacancies, and also the expected benefits for outsiders (floaters) of joining a group, affect selection on floaters to force their way into an established group. At the same time, we examine (ii) how the same demographic processes, and also the expected fitness costs of sharing their territory with a potential entrant, affect selection on territory owners (insiders) to resist the joining effort of outsiders. In any such conflict over group-membership we also consider possible power asymmetries between insiders and outsiders. Following from (i) and (ii) we derive (iii) the expected degree of group-living (communal breeding) in the population.

## The Model

The present model is based on a demographic scenario described in detail in Port et al. (2011). In brief, we consider an infinite island population structured into territories of equal quality. Each territory contains two breeding sites, each of which can be either empty or occupied by a single breeder of the focal sex. Note that, like previous demographically explicit models of social evolution (Pen and Weissing 2000; Kokko and Lundberg 2001; Kokko and Ekman 2002; Hamilton and Taborsky 2005; Port et al. 2011) we consider group formation among same-sex individuals rather than between conspecifics more generally (Giraldeau and Caraco 1993; Higashi and Yamamura 1993).

We model population dynamics in continuous time. We assume that residents on territories where both breeding sites are occupied (hereafter referred to as communal breeders) experience a baseline mortality rate of *m*_R_. Residents on territories where only one site is occupied (hereafter referred to as lone breeders) experience a mortality rate of *m*_L_ (owing to the possible costs of territory defense for lone breeders *m*_L_ ≥ *m*_R_, see below). On territories where at least one breeding site is occupied, offspring are produced one at a time at rate *F* (note that this is the rate of offspring production per territory, not per breeder). We assume that, on communal territories, reproduction is unevenly shared among co-breeders, with subordinate breeders having a probability *p* (*P* ≤ 0.5) of siring the single offspring produced each breeding bout, and dominants having a probability 1-*p*. Offspring disperse from their natal territory to join a pool of floaters where they compete with same-sex individuals over breeding vacancies. Assuming an even sex ratio at birth and no sex-biased mortality, half the floaters are of the focal sex. Floaters die at rate *m*_F_ and discover territories at rate *e* (Table 1).

**Table 1:** List of symbols

Symbol	Definition
*m*_*R*_	Baseline mortality rate of residents
*m*_*L*_	Mortality rate of lone breeders
(*m*_Fb_), *m*_*F*_	(Baseline) mortality rate of floaters
*F*	Rate of fecundity per territory
*p*	Subordinate breeder's share of reproduction on communal territory
*e*	Rate at which floaters discover new territories
*P*_join_	Probability with which floaters join established (lone) breeders
*j*	Floaters' effort to join lone breeder
*r*	Lone breeders' effort to reject joining attempts of floaters
*b*	Power asymmetry between residents and floaters
*c*	cost exponent relating effort invested into conflict to mortality costs
*q*_0_, *q*_1_, *q*_2_	Proportion of empty territories, lone breeder territories, and communal territories, respectively, in the population.
*δ*_*F*_	Density of floaters in the population

We assume that whenever a floater discovers an empty territory it simply occupies one of the two breeding sites and turns into a lone breeder. When a floater discovers a lone breeder territory, it may attempt to occupy the remaining breeding site (dependent on whether doing so is more beneficial than remaining in the floater pool). However, because in the present model lone breeders do not benefit from sharing their territory, they should try to repel the intruder. Thus, if a floater benefits from joining, a conflict over group membership ensues. In any such conflict the probability of a floater joining a lone breeder, *P*_join_, is given by the following equation (the contest success function; Cant 2012):


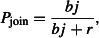
(1)

where *j* is the effort invested by the floater to join the group, *r* is the effort invested by the lone breeder to reject the floater, and *b* is the power asymmetry between floater and resident. We assume that, owing to the ownership advantage (Maynard-Smith and Parker 1976; Leimar and Enquist 1984) and/or superior resource holding potential (RHP, Parker 1974) of residents, *b* < 1, that is, residents are always stronger competitors than floaters. As a consequence, if the floater succeeds entering the lone breeder's territory, it will initially take on the role of a subordinate breeder on a communal territory, while the former lone breeder will take on the role of a dominant breeder. For simplicity, we assume that a floater cannot take over the territory by evicting the former resident and become a lone breeder itself.

The form of the contest success function used in the present model (eq. 1) is the most appropriate form for the biological problem under consideration, because it requires individuals to invest at least a minimal amount of effort if they were to gain any benefit (see Cant 2012 for discussion). In the present model, if floaters benefit from group-living, they have to put in some joining effort, or else they will not gain group membership.

We assume that effort invested in the struggle over group-membership has a negative impact on an individual's survival. The magnitude of this impact depends on the amount of effort invested in each struggle (*j* or *r*) as well as on the rate at which such struggles occur. For instance, a lone breeder pays a cost proportional to its defensive effort *r* each time it encounters a floater trying to join its territory, and such encounters occur at a rate equal to the density of floaters in the population (*δ*_F_*,* an evolving parameter of our model, see below) multiplied by their territory exploration rate (*e*). In sum, the mortality rate of lone breeders (*m*_*L*_) is equal to the baseline mortality of residents (*m*_*R*_) plus an additional component owing to the lone breeder's defensive effort:


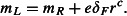
(2a)

Likewise, the mortality rate of floaters is equal to the baseline mortality of floaters (*m*_Fb_) plus a component owing to the floater's joining effort:


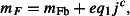
(2b)

where *q*_1_ represents the proportion of lone breeder territories in the population (again, an evolving parameter, see below). In equations 2 and 3, *c* is a cost exponent which relates the effort invested into the conflict to the mortality costs it imposes. If *c* = 1, costs increase linearly with effort, if *c* > 1, small levels of effort (e.g., territorial displays) incur relatively low costs, whereas larger levels of effort (e.g., fights) incur relatively high costs.

We want to determine the evolutionarily stable levels of floater joining effort (*j*) and lone breeder rejecting effort (*r*). To find stable levels of *j* and *r*, we use an approach similar to many recent analyses of social behavior in structured populations (Gardner and West 2006; Alizon and Taylor 2008; Johnstone and Cant 2008). For a monomorphic population characterized by given values of *j* and *r*, we first determine the frequencies of different patch types at demographic equilibrium (step 1 below). We then determine the fitness of different classes of individual at demographic equilibrium (step 2). Finally, we examine the effect of a rare mutation of small effect (causing a slight change in the values of *j* and/or *r*) on the fitness of individuals bearing the mutant allele (step 3).

### Demographic dynamics and equilibria

A patch can change from an empty territory (in which none of the breeding sites is occupied) into a lone breeder territory and vice versa*,* or from a lone breeder territory into a communal territory and vice versa. A transition between two types of patch occurs whenever one of the following events occurs: (i) an existing breeder dies, or (ii) a new breeder joins the patch. We can thus write for the rates of change in the frequencies of the three types of patch


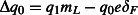
(3)


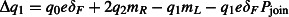
(4)


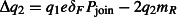
(5)

where *q*_0_, *q*_1_, *q*_2_, and *δ*_*F*_ represent the proportion of empty territories, lone breeder territories, communal territories, and the density of floaters, respectively. For instance, the proportion of lone breeder territories (eq. 5) increases whenever a floater discovers an empty territory (at rate *q*_0_*e* per floater), or when a breeder on a communal territory dies (at rate *m*_*R*_ per breeder per communal territory). The proportion of lone breeder territories decreases whenever a lone breeder dies (at rate *m*_L_ per lone breeder) or when a lone breeder is joined by a floater (at rate *q*_1_*eP*_join_ per floater).

Floaters are added to the floater pool through dispersing offspring of the focal sex, and they are lost from the floater pool whenever a floater dies, discovers an empty territory, or manages to join a lone breeder. We can thus write for the rate of change in floater density (*δ*_*F*_):


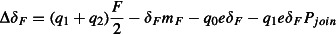
(6)

At demographic equilibrium, both the proportion of patch types and the density of floaters must not change, hence


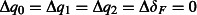
(7)

Simultaneously solving equation 8 subject to the constraint *q*_0_ + *q*_1_ + *q*_2_ = 1 gives the proportion of empty territories (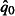), lone breeder territories (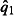), communal territories (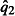), and the density of floaters (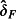) at demographic equilibrium. We regard only combinations of parameter values that yield a viable and stable population. Because the relevant expressions are cumbersome we do not give them here.

### Fitness expressions

We use lifetime reproductive success as our measure of fitness. The expected future fitness of an individual in each of the four possible states (floater, lone breeder, subordinate, dominant) depends on its rate of offspring production while in that state as well as on the rates of all possible transitions out of that state. For instance, a lone breeder (eq. 9, below) can die (at rate *m*_*L*_) in which case it obtains zero fitness, or it can reproduce (at rate *F*), in which case it obtains one fitness unit immediately (corresponding to the one offspring produced each breeding bout) and remains in the state of a lone breeder with expected future fitness gains of *W*_*L*_. Finally, it can be joined by a floater (at rate *eδ*_*F*_*P*_join_), in which case it turns into a dominant breeder on a communal territory with expected future fitness gains of *W*_*D*_. In a continuous time model, the probability of any one of these events occurring before any other is given by the rate at which it is expected to happen relative to the sum of the rates of all possible events. The fitness of lone breeders (*W*_*L*_), dominants (*W*_*D*_), subordinates (*W*_*S*_), and floaters (*W*_*F*_), respectively, can thus be written as follows:


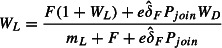
(8)


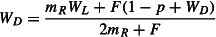
(9)


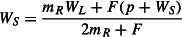
(10)


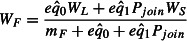
(11)

In equations (8)–(11), 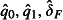 represent the proportion of empty territories, lone breeder territories, and the density of floaters, respectively, in the population at demographic equilibrium, as calculated in step 1. Simultaneously solving expressions (8–11) yields the fitness of the four classes of individuals at evolutionary equilibrium. Because we cannot derive analytical solutions, we use a numerical procedure, which is described in the Appendix.

### Solving the model

To determine stable levels of conflict over group membership and the resulting population level of communal breeding, we need to find the stable levels of *j* and *r* given the set of parameters defined in our model (*m*_*R*_*, m*_Fb_*, c, F, e, b, p*). Note that *m*_L_ and *m*_F_, as well as the proportion of territories (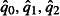), and the density of floaters (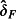) at demographic equilibrium are evolving properties of our model, which depend themselves on *j* and *r*.

To find the stable levels of *j* and *r*, we use an adaptive dynamics approach (Geritz et al. 1998): we are interested in whether a monomorphic population characterized by given values of *j* and *r* can be invaded by an initially rare mutant of small effect, that is, a mutant expressing a slightly different value j* and/or r*. Because any change in *j* is expressed only by floaters, we can determine the effect of a small mutation with respect to *j* (j*) by deriving the selection gradient ∂*W_F_/∂*_*j*_ at *j* = j*, weighted according to the density of floaters in the population:


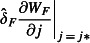
(12)

Likewise, because any change in *r* is expressed only by lone breeders, we can determine the effect of a small mutation with respect to *r* (r*) by deriving the selection gradient *∂W*_L_*/∂*_r_ at *r* = r*, weighted according to the proportion of lone breeder territories:


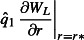
(13)

Because, we cannot solve expressions (12) and (13) analytically (i.e., by setting the selection gradients equal to zero and solving for *j* and *r*) we use an iterative, numerical solution procedure, which is described in detail in the Appendix.

## Results

We derive stable levels of joining effort *j* and rejecting effort *r* for varying levels of key parameters specified by our model. First, we are interested in how *j* and *r* are affected by demographic factors, more specifically, by the rate of per-group fecundity (*F*) relative to resident mortality (*m*_R_). The rate of fecundity (relative to *m*_R_) determines the degree of habitat saturation, and hence, competition among floaters for breeding vacancies as well as the costs of territory defense for residents. Second, we are interested in how *j* and *r* are affected by within-group conflicts, reflected in the share of reproduction (*p*) floaters can expect by joining a group. Finally, we explore how *j* and *r* are affected by power differences between insiders and outsiders (*b*).

### Demographic factors

**Figure 1:**
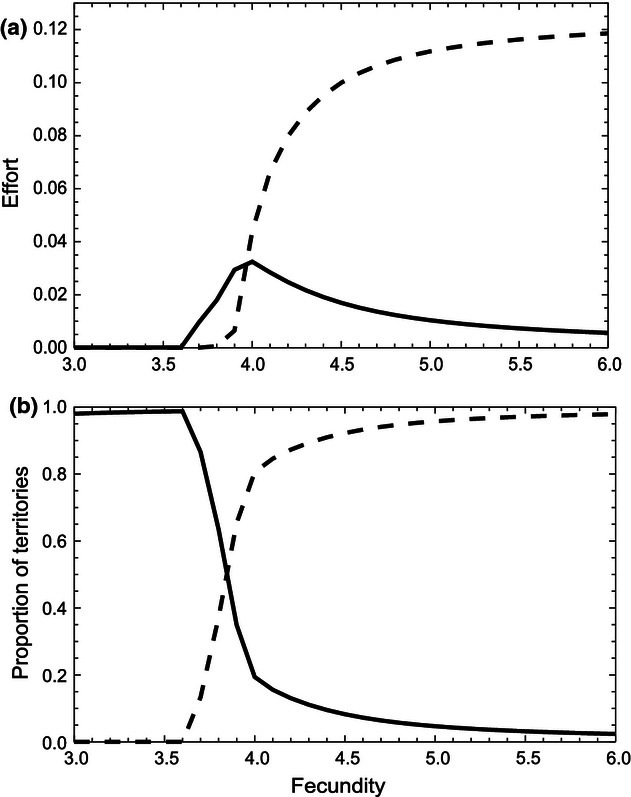
(A) The stable levels of joining effort (*j*, dashed line) and rejecting effort (*r*, solid line) as a function of per-group fecundity (*F*). (B) The proportion of lone breeder territories (solid line) and communal territories (dashed line) in the population at evolutionary equilibrium. In both panels fecundity is defined as the rate of fecundity per group, relative to the mortality rate of a resident (*m*_R_), which is set to 1. Other parameters are set as follows: *m*_Fb_ = 1, *c* = 2, *e* = 100, *b* = 0.75, *P* = 0.1.

As long as per-group fecundity (*F*) is not much higher than resident mortality (*m*_R_), we find that *j* ≍ 0, because floaters do not benefit from joining a group (Fig. 1A). In this case, as the turnover rate of territories is sufficiently high, outsiders are better off waiting in the floater pool for a vacancy to arise than trying to compete with an already established breeder on that breeder's territory. But as fecundity increases, it becomes more beneficial for floaters to take on the role of a subordinate than to remain in the floater pool. Consequently, a conflict over group membership ensues, in which the amount of effort (*j*) floaters invest to gain group membership rapidly increases as fecundity further increases and the competition for breeding vacancies gets more intense (Fig. 1A). Because residents, on the other hand, do not benefit from sharing their territory, they are selected to also raise their rejecting effort (*r*) in order to counter the floater's joining attempt. However, a resident's stable rejecting effort drops again as fecundity further increases (Fig. 1A). The reason is that if fecundity is much higher than resident mortality, the density of floaters is high and residents are frequently encountered by potential joiners. In this situation, territory defense becomes too costly and residents are selected to reduce their rejecting effort, in other words, to give in to the intruder pressure and allow outsiders to join. As soon as a conflict over group membership ensues, the population gradually shifts from singular breeding to communal breeding (Fig. 1B). Once residents have reduced their rejecting effort to very low levels, the population almost entirely consists of communal breeders.

**Figure 2:**
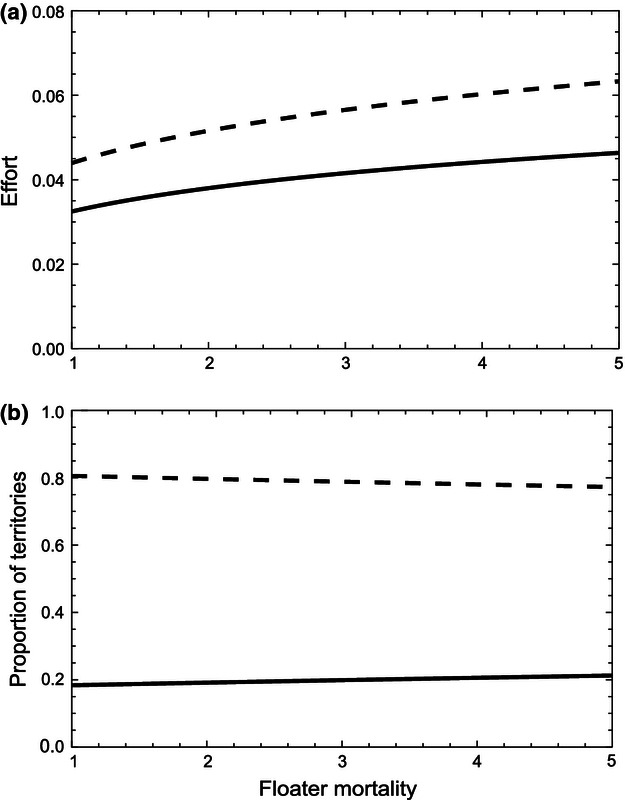
(A) The stable levels of joining effort (*j*, dashed line) and rejecting effort (*r*, solid line) as a function of floater mortality (*m*_Fb_). (B) The proportion of lone breeder territories (solid line) and communal territories (dashed line) in the population at evolutionary equilibrium. In both panels floater mortality is defined as the rate of mortality per floater, relative to the mortality rate of a resident (*m*_*R*_), which is set to 1. Other parameters are set as follows: *F* = 4, *c* = 2, *e* = 100, *b* = 0.75, *P* = 0.1.

In contrast to the marked effect of fecundity (*F*, relative to *m*_R_) the mortality of floaters (*m*_Fb_, relative to *m*_R_) exerts little influence on the resolution of conflicts over group-membership. Even though floaters benefit more from joining if floater mortality is high, high floater mortality decreases the density of floaters in the population. As a result, individual floaters face less competition for group-vacancies, rendering selection on *j* only weakly affected by floater mortality (Fig. 2A). Moreover, at lower floater densities territory defense becomes less costly for residents, which can therefore maintain relatively high levels of resistance. As a result of both aforementioned effects (weak selection on *j*, and decreasing costs of defense with increasing *m*_Fb_), the probability of group-formation is largely unaffected by floater mortality. In effect, owing to lower encounter rates, communal breeding tends to be less common if floater mortality is high, Fig. 2B. This result confirms a previous result derived for cooperative breeders (Pen and Weissing 2000), showing that dispersal related mortality only weakly affects the evolution of group formation.

### Within-group conflict

**Figure 3:**
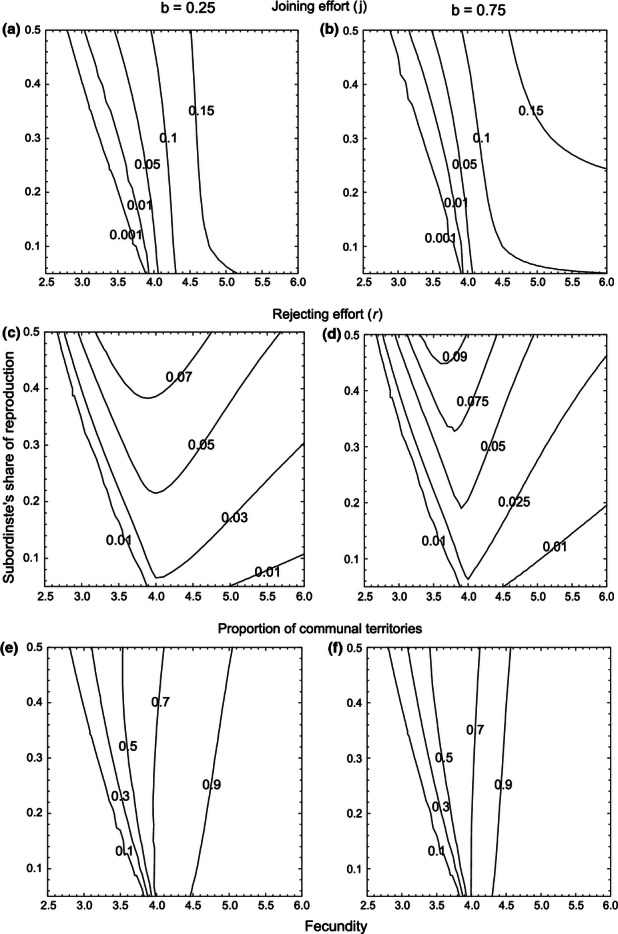
Stable joining effort (*j*, A, B), rejecting effort (*r*, C, D), and the resulting proportion of communal territories in the population (E, F) at various levels of per group fecundity (*F*) and reproductive skew (*p*). Lines represent isoclines indicating different levels of *j* (A, B), *r* (C, D) and the proportion of communal territories (E, F). In (A), (C), and (E) *b* = 0.25, whereas in (B), (D), (F) *b* = 0.75. All other parameters are set as follows: *m*_*R*_ = 1, *m*_*Fb*_ = 1, *c* = 2, *e* = 100.

If outsiders can expect to gain more from joining a group, they are selected to invest more effort in order to reach their goal. Figure 3A and B shows this relationship with respect to the share of reproduction (*p*) an outsider can expect as a subordinate breeder on a communal territory. As *p* increases, outsiders invest more effort into the struggle over group membership. This effect is weak, however, compared with the effect of floater density (fecundity). Moreover, if outsiders gain more from joining, they also impose higher costs on residents. Thus, residents, as well, are selected to invest more strongly into the conflict over group membership (Fig. 3C and D) and the increased rejecting effort of insiders counterbalances the increased joining effort of outsiders. As a consequence, the level of reproductive skew has only a weak effect on the probability of group formation (Fig. 3E and F). Note that at low densities (low *F*) communal breeding is slightly more common if reproductive skew is low whereas, at high densities, it is slightly less common if skew is low, but in either case, this effect is weak compared with the effects of demographic parameters.

### Power asymmetries

Group formation is also only marginally influenced by power asymmetries between insiders and outsiders (*b*). Even though residents need to put in more effort to reject a stronger competitor (compare Fig. 3C and D) they can afford this effort as long as floater density is low. As a result, the level of communal breeding is virtually the same, irrespective of whether *b* is high or low (Fig. 3E and F). At high densities, the elevated costs of rejecting a stronger competitor weigh more than at low densities. Residents, therefore, reduce their rejecting effort more strongly against strong competitors (Fig. 3C and D), leading to slightly higher levels of communal breeding if *b* is high (Fig. 3E and F). The power asymmetry between insiders and outsiders thus acts in concert with population dynamics, but its overall effect is weak compared with the effect of floater density per se.

### Changing our assumptions

Above, we have considered a subordinate's share of reproduction as fixed benefit of communal breeding for subordinates. Yet it could be argued that it depends on the initial power asymmetry between the dominant/resident and the former floater, for instance, if *b* represented an inherent difference in RHP between insiders and outsiders, rather than a property of the territory or ownership advantage. In such a case, if *p* is a function of *b*, one might expect an overall larger effect of *b* on group formation. This is not the case, however. Even though stronger outsiders, in this case, benefit more from joining, they also impose higher costs on residents. As a consequence, residents increase their rejecting effort and the proportion of communal breeding does not change significantly (M. Port, unpublished results).

## Discussion

We have looked at the evolution of group-formation among non-relatives, focusing on cases in which group-membership is disputed between insiders and outsiders. Our model reveals that whether or not groups form is mainly determined by vital rates of residents (fecundity and mortality) and the resulting population dynamics. By contrast, the power difference between insiders (residents) and outsiders (floaters), the dispersal related risk of mortality, but also the share of reproduction outsiders can expect to gain within a group, exert a comparatively minor influence on the process of group formation. Given the recent emphasis on reproductive skew in social evolution theory, this latter result is perhaps surprising. It will be examined in the next paragraph. We will then shift our attention to population level processes and discuss empirical applications of our model. Finally, we will examine the relationship between our model and models of territorial conflict and highlight avenues for future research.

Over the past two decades, a prominent group of theoretical models have suggested that the resolution of within-group conflict over reproduction plays a crucial role in determining the stability of social groups of animals (models of reproductive skew, reviewed in Johnstone 2000; Nonacs and Hager 2010). One criticism of these models, however, is that they generally treat the options available to individuals outside their groups as fixed (Cant and Johnstone 2009; Port and Kappeler 2010). By contrast, in the present model, the expected payoff to a floater who fails to secure a place as a subordinate is not extrinsically specified, but emerges from the dynamics of the population. It is this difference that accounts for the comparatively weak influence of skew on group formation in our analysis. Any change in skew leads to a change not only in the payoff that a floater stands to gain from forcing entry into a given group, but also in the payoff that it can expect to gain elsewhere, that is, by trying to force entry into a different group. The degree of reproductive skew within groups only exercises a significant influence on conflicts over group-membership if floater densities are low and vacant territories correspondingly common, as in this case the payoff to looking elsewhere consists mostly in the chance of finding an empty territory, which is independent of skew. We need to stress, however, that our model is built on the assumption that all territories are of equal quality (with equal levels of reproductive skew). It would thus be an interesting avenue for future research to relax this assumption and to introduce a greater degree of heterogeneity in patch quality. Nonetheless, our results indicate that the division of reproduction within groups has a much weaker effect on group formation and stability than previously thought, and they also show that this effect is largely overridden by population dynamics. In light of this result, it is not surprising that empirical studies have so far failed to uncover significant effects of reproductive skew on the stability of social groups of animals (Queller et al. 2000; Clutton-Brock et al. 2001; Port et al. 2012).

The most important result emerging from our model is that, owing to the costs of territory defense, residents are most likely to give in to the joining attempts of floaters when intruder pressure, that is, the number of outsiders, is high. This result adds significantly to the existing literature on insider–outsider conflicts (Pulliam and Caraco 1984; Giraldeau and Caraco 1993; Higashi and Yamamura 1993). Preliminary support comes from a comparison of two populations of Verreaux's sifaka (*Propithecus verreauxi*), a lemur living in small multi-male, multi-female groups. In this species, males do not cooperate and there are no benefits for males of sharing their territories with other males (Port et al. 2012). Nonetheless, in a well-studied population in western Madagascar, about one-third of groups contain more than one immigrant male (Kappeler et al. 2009; Port et al. 2012). Moreover, in another population from south-west Madagascar, where these lemurs occur at overall higher population densities, the average number of males per group is even higher (Richard et al. 2002; Lawler et al. 2003). More generally, many groups of primates contain several unrelated males, and in most cases there are apparently no synergistic benefits of forming such associations (Kappeler 2000). Our model helps to explain why multi-male groups of primates form and why the number of males can differ significantly between populations and species.

There are many more species of vertebrates in which several unrelated individuals can share a communal territory, examples include several species of cichlid fish (Taborsky 2001), communally breeding birds such as guira cuckoos (*Guira guira*, Quinn et al. 1994), or Taiwan yuhinas (*Yuhina brunneiceps*, Shen et al. 2012), as well as species with facultatively polygyneous or polygynandrous mating systems such as dunnocks (*Prunella modularis*, Davies 1992), pukeko (*Porphyrio porphyrio*, Jamieson 1997), or waterbuck (*Kobus ellipsiprymnus*, Wirtz 1981). In most of these species, the precise relationship between the demographic factors emphasized by our model and the type of social system warrants further examination. Moreover, there are perhaps even more species of ‘classic’ cooperative breeders, in which groups are formed via natal philopatry (Clutton-Brock 2002; Koenig and Dickinson 2004), but in which conflicts over group-membership may be equally important (see e.g., Cant et al. 2010). Theoretical models examining this type of social system have so far assumed implicitly that one party is in complete control of group-membership (Pen and Weissing 2000; Kokko and Lundberg 2001) and it would be interesting to incorporate the present approach of conflict resolution into those models.

It is worth comparing our analysis with a recent demographically explicit model of territoriality (Kokko et al. 2006). In accordance with our results, the latter model also found that differences in RHP have a fairly small effect on the outcome of territorial fights. However, we found that territory owners decrease their defensive effort as floater density increases, whereas Kokko et al. (2006) found that owners maintained high levels of aggressiveness even at high floater densities (but note that Kokko et al. modeled the probability of a conflict occurring in any encounter, whereas we looked at the effort invested in any such encounter). It is thus worthwhile to stress an important difference between these models: in Kokko et al.'s model floaters always try to take over territories (evicting or killing the established owner) whereas in our model they never do (but instead join the established owner as a subordinate). Clearly, therefore, if everything is at stake for residents, they should never give in to floaters and should continue fighting even at high floater densities. This comparison highlights a fruitful avenue for future research: future modeling could provide floaters with both strategic options and could examine how they allocate their effort, that is, whether they opt for taking over a territory or for joining an established resident in the role of a subordinate. In this way, an important but so far largely ignored question could be resolved, namely which factors determine the transition between strict territoriality (where individuals fight over the sole possession of territories) and group-living? Moreover, the present model is restricted to associations of only two residents and it also excludes the possibility for cooperation among co-residents. If floaters were given the option to join at a lower position of the breeding-queue and if co-residents were further given the option to cooperatively defend their territory against such joining attempts, it could also be examined if, and at what point in the aforementioned transition, cooperation can evolve. Models of territoriality and of social evolution have so far been developed in isolation from one other. Above, we have highlighted a possible way of synthesizing them in order to provide a more comprehensive theory of social evolution.

## APPENDIX

To solve our model for stable joining efforts (*j*) and rejecting efforts (*r*), we use an iterative, numerical solution procedure. We start by considering a population with an arbitrary, given combination of *j* and *r*. In each time-step of the iteration we then proceed as follows: (i) we determine the proportion of territories and the density of floaters at demographic equilibrium (step 1, main text); (ii) using Mathematica 7.0, we numerically solve equations (8)–(11) to determine the fitness of the different classes of individuals at demographic equilibrium (step 2, main text); (iii) we solve expressions (12) and (13) numerically to determine the direction and strength of selection with respect to *j* and *r* (step 3, main text); (iv) we update the population (resident) values of *j* and *r* by adding to each the value of the relevant selection gradient, multiplied by a coefficient which decreased over successive iterations (so that we impose greater damping as the equilibrium is approached). We repeat steps (i)–(iv) until both selection gradients <10^−5^ or until either *j* or *r* ≍ 0 (10^−10^) and the remaining selection gradient <10^−5^. The combination of *j* and *r* obtained in this way is the singular strategy combination . Starting values used in our iterations were [*j*, *r*]_*t* = 0_ = [0.1, 0.1]. Other starting values merely altered the time until convergence was reached but did not affect the equilibrium values of *j* and *r* significantly.
